# Meat-Inspection

**Published:** 1900-02

**Authors:** S. G. Burkholder

**Affiliations:** Chicago, Ill.; Professor of Meat-Inspection in the M’killip Veterinary College


### MEAT-INSPECTION.

By S. G. Burkholder, M.D., V.S.,
CHICAGO, ILL.
PROFESSOR OF MEAT-INSPECTION IN THE M'KILLIP VETERINARY COLLEGE.

(Continued from page 13, January, 1900.)

I WILL not take up any more time in discussing these different infectious diseases, with which you are all familiar, but will call your attention to the methods of animal- and meat-inspection at the various stockyards and packing-houses, as conducted by the bureau at the present time.

Both ante-mortem and post-mortem inspection is made of all cattle, sheep and hogs arriving at the various stockyards and packing-houses where Federal inspection is desired.

The ante-mortem inspection is quite thorough, its principal object being to prevent a spread of infectious diseases by animals that are again shipped to the country as feeders and breeders. Those condemned in the yards are tagged and later slaughtered by the permission and under the immediate supervision of inspectors of the bureau, and disposed of accordingly.

Hogs are condemned on ante-mortem inspection for cholera, advanced pregnancy, extensive and suppurating wounds, special tumors and various sporadic febrile diseases. Cholera is the most important.

Sheep are condemned for emaciation, tumors, suppurating wounds, and the various febrile, infectious and parasitic diseases. Sheep-scab is the most important. No sheep, after once entering the yards (at least in Chicago), are allowed to leave again alive, either for export or interstate trade, until they are dipped, whether they show symptoms of scabies or not. This, you can readily appreciate, is a very thorough and wise precaution.

Cattle are condemned on ante-mortem inspection for the same general diseases for which sheep and hogs are condemned, as well as for the various infectious diseases bovine flesh is heir to.

Cattle for export are reinspected, and a record is kept showing from what part of the country each herd came. If there should be an outbreak of some infectious disease among a herd the disease could be immediately traced to its spreading focus and suppressed.

Thus, you see, the ante-mortem inspection at the stockyards is quite thorough and very practical. The post-mortem inspection of the carcasses of the slaughtered animals at the packing-house is equally thorough. Carcasses are condemned for all abnormal conditions which prove to be generalized, presenting lesions that markedly interfere with either nutrition or elimination, and all advancing inflammatory and infectious diseases. Pregnancy is apparently almost entirely overlooked.

The question often arises, Where shall we draw the line, especially in such diseases as cancer, actinomycosis, tuberculosis, etc.?

Regarding cancer we know very little. We know that it exists, and occasionally see very suspicious neoplasms in the various animals. Reports from England show that a large number of their cattle suffer from angry-looking, nodular growths, located near the lips or on the throat of these animals. The malignancy of these growths has not been proven, but they look very suspicious and are at least deserving of an investigation by competent pathologists. Several members of the English Parliament are interested in the subject, and will, no doubt, have the matter brought before that body at an early date.

American cattle are very apt to suffer from angry-looking tumors, apparently originating from the membrana nictatans. These tumors grow very rapidly, involving the surrounding tissues, filling the orbit and finally destroying the eye. This growth presents the histological picture of a true epithelioma, and presents the usual disseminating characteristics. The neighboring lymphatics are invariably involved, showing its progressive and metastatic nature.

It is undoubtedly a true cancerous growth; but the subject has hitherto been neglected, and its positive nature will not be known until some one interested in this line of work shall make a thorough investigation. The material to work on is abundant, and the veterinary profession has no excuse for lagging so far behind its sister profession in the knowledge of the nature and histopathology of many of these pathological conditions found in animals.

The knowledge of the exact causes and nature of cancer in the human being is not yet established. We do, however, know that the nnmber of people who die each year from this disease is enormous and that it increases in prevalence with amazing rapidity. Prof. Roswell Park makes the startling prophecy that if for the next ten years the present relative death-rates are maintained, in 1909 there will be more deaths in the State of New York from cancer than from consumption, smallpox and typhoid fever combined.

How can we account for the lamentable propagation of this malignant monster? Might not cattle, whose products form a large part of our diet, be transmitting agents? This question has thus far received little attention. It is a question, however, that should be thoroughly investigated, and the veterinary and medical professions should coöperate in this matter, and lose no time in probing into the mysteries of the source of this death-dealing agent. Cancer to-day is one of the most fatal, most malignant, and most prevalent diseases among mankind, yet nothing has been accomplished to check its progress.

Regarding actinomycosis and tuberculosis there seems to be a difference of opinion. We know that both are transmissible from one animal to another, and also to man. At any rate, cases of actinomycosis occurring in man are recorded. It is apparently the same disease that we find in cattle, as the fungus is identical.

Dr. Edward Moore, a prominent veterinary surgeon of Albany, N. Y., recently published a long article in the *New York Medical Journal*, claiming that bovine tuberculosis is not transmissible to man, and *vice versa*, and that the bacillus has become so modified by *habitat* as to become specifically distinct. His views are supported by Drs. Theobald Smith, Cooper Curtice and others. This difference of opinion simply proves that our knowledge concerning this disease, or rather its transmission from animals to man, is still somewhat vague, and that a more thorough study is evidently necessary to establish conclusive evidence regarding the intra-specific transmission of tuberculosis between man and cattle.

Accepting, however, the possibility of transmitting both these diseases from animals to man, the question arises, Where should the meat-inspector draw the line? Opinion seems to differ in this respect. It is safe to say, however, that after either one of the above diseases is developed to such a degree that it can be diagnosed with a certainty in the living animal it is very risky to use at least the milk from such animals. They should be immediately killed, carefully inspected by competent inspectors and properly disposed of.

The first thing to be looked for in the post-mortem inspection is the focal or primary lesion. If this can be discovered and the neighboring lymphatics are involved, the specific material has disseminated and the disease might be considered general, although no pathological changes in the remote lymphatics are noticeable. After the infective material once enters the lymphatic circulation, the disease is no longer definitely circumscribed.

It is claimed that fluid amounting to from 3 to 8 per cent. of the body weight flows into and is absorbed from the peritoneal cavity every hour. This fluid is thrown out and again taken up by the lymphatics. This gives us an idea of the immense volume of fluid circulating through the system, and how easy it is for germs to gain access to the lymphatic circulation and be disseminated throughout the system. Some of the germs are caught in the meshes of the lymphatic glands through which the fluid flows and is filtered in its passage to the venous circulation. This, then, is the reason why we first notice the neighboring lymphatics affected whenever there is an extension from a local infection. Some germs escape the lymphatic glands and pass into the venous circulation with the lymph-stream. Either from the hæmic or lymph circulation the germs find their way into various excretory and secretory glands, where they may lodge and form a secondary lesion, be killed by the activity of the gland cells or leucocytes, or be thrown out with the secretions of the gland. Thus we find that pyogenic germs in a septic peritonitis are carried to all parts of the body, and are found in the bile, saliva, milk, and other secretions.

It was formerly supposed and is still held by some that if the mammary glands in tuberculous cattle are free from tubercular deposits, the milk does not contain tubercle bacilli. This we know to be a fallacy. It is a well-known fact that tubercular deposits are seldom if ever found in voluntary muscular tissue, yet it is possible to transmit tuberculosis by inoculating guinea-pigs with the myosin expressed from the muscular tissue of tuberculous cattle. This, then, proves that it is hard to draw a distinct line between a strictly local infection and a more or less general infection.

The local inflammation produced by infective germs is an effort of nature to confine the infective material, in which it sometimes succeeds, thus preventing diffusion and general dissemination. Now, these general principles hold true in all infectious diseases of the domestic animals, and the same rule should apply to all.

Regarding tuberculosis, I consider there is more danger from the use of the milk from infected cattle than from the meat, because meat is usually subjected to the process of cooking before it is used as food, while the milk is so universally used as a food without first boiling it, especially by the sick and infants, in whom the power of resistance is lowered and the destructive influence of the gastric juice is lacking.

The importance of the knowledge of the source of all animal products, a thorough meat-inspection by competent inspectors and the proper disposition of diseased and contaminated products appeals to every intelligent citizen.

In the first part of my paper I have briefly reviewed part of the work that has been accomplished by the Bureau of Animal Industry, which, as you will agree, reflects great credit upon the members of this body. I do not wish to criticise the operations of the bureau, but in my opinion its work in certain lines might be improved.

Every member of the veterinary profession rather painfully appreciates the fact that our knowledge of animal pathology is as yet at a very low ebb. Why is it? It is not due to a lack of material, for in this respect we have rather the advantage of our sister profession. I am of the opinion, however, that this lack of advancement in this most important study is not due to a want of ability and interest on the part of the heads of the bureau, but rather to the lack of funds. The amount of interesting pathological material that goes to waste every year at our stockyards is immense, and can only be appreciated by one who has been in the service. No place in the United States furnishes the same amount of material for pathological research. It is true there is a pathological division in the bureau, operated by competent men, but their force is small, and being located in Washington, D. C., it is impossible for them to do the work justice. What we need is a pathological laboratory right here in Chicago, where material is abundant, and every facility provided for scientific research. Such a procedure would necessarily require an addition to the present amount of appropriation granted to the bureau. This can only be obtained through legislative enactment.

It becomes the duty, then, of all members of the veterinary profession to emphasize the importance of such a procedure to their respective Representatives in Congress, as well as encourage united action by the various veterinary organizations. In this way we can elevate the standard of the veterinary profession equal to that enjoyed by the veterinary profession of the European countries.

I hope that the members of the profession will appreciate the importance of thus endeavoring to improve our knowledge of the nature of animal diseases, thereby enabling us to render better services to the people in the way of protecting them from contracting various diseases from the products of animals as well as preventing the spread of diseases among animals, and the consequent loss of large amounts of money to the stock-raisers.

